# Opportunities, obstacles and challenges of nano-immunotherapy in melanoma

**DOI:** 10.3389/fimmu.2025.1611423

**Published:** 2025-08-08

**Authors:** Zexing Shan, Fei Liu

**Affiliations:** ^1^ Department of Gastric Surgery, Liaoning Cancer Hospital and Institute, Cancer Hospital of China Medical University, Shenyang, China; ^2^ Department of Bone and Soft Tissue Tumor Surgery, Cancer Hospital of China Medical University, Liaoning Cancer Hospital & Institute, Shenyang, China

**Keywords:** nano-immunotherapy, melanoma, tumor microenvironment (TME), immune checkpoint inhibitors (ICIs), nanoparticles

## Abstract

Melanoma is an exceptionally aggressive form of skin cancer, and its prognosis becomes dire once it metastasizes. Although substantial progress has been made in the field of immunotherapy, significant hurdles such as tumor cell immune evasion, the tumor microenvironment (TME), and immune-related adverse effects persist. Recent advancements in nanotechnology offer promising solutions to these challenges by enhancing targeting, stability, and delivery of immunotherapeutic agents. Nano-immunotherapy, which synergizes nanotechnology with immunotherapy, is evolving into a groundbreaking approach for melanoma treatment. Various nanoparticles, including liposomes, dendrimers, and polymeric nanoparticles (PNPs), are under investigation to boost immune responses, deliver immune checkpoint inhibitors (ICIs), and modulate the TME. These nanoparticles can be engineered for precise drug delivery, minimizing off-target effects and enhancing therapeutic outcomes. Moreover, the encapsulation of sensitive molecules such as cytokines, vaccines, and antibodies within nanoparticles ensures their stability and bioavailability. This review delves into the recent advancements in nano-immunotherapy for melanoma, emphasizing the mechanisms through which nanoparticles enhance immune activation and counteract the immunosuppressive TME. Additionally, we address the challenges of translating these nanomaterials into clinical settings, including optimizing nanoparticle design, ensuring safety, and achieving robust immune activation. This review provides a detailed examination of the current landscape and future potential of nano-immunotherapy as a promising strategy for melanoma treatment.

## 1 Introduction

Melanoma, an aggressive form of skin cancer originating from melanocytes, is often driven by genetic mutations, particularly those induced by environmental factors like ultraviolet (UV) radiation (1–3). This malignancy has a high metastatic potential and is considered a major cause of skin cancer-related mortality worldwide (4, 5). The incidence of melanoma is increasing, especially among younger populations, presenting a critical public health challenge (6, 7). Despite advancements in early detection and treatment, the prognosis for patients with advanced or metastatic melanoma remains poor, underscoring the need for novel and more effective therapeutic strategies (8, 9). Melanoma’s high mutational burden and the presence of multiple neoantigens enhance its immunogenicity, making it an ideal target for immune-based therapies, though it is complicated by immune evasion mechanisms within the tumor microenvironment (TME) (10). Recent advancements in immunotherapy have significantly improved the treatment landscape for melanoma, particularly with immune checkpoint inhibitors (ICIs) targeting PD-1, PD-L1, and CTLA-4 (11–13). These therapies have yielded promising results, especially in patients with advanced disease (8, 10). However, challenges such as primary and acquired resistance, immune evasion, and the immunosuppressive TME continue to limit the effectiveness of these treatments. These hurdles highlight the need for novel strategies that can enhance immune activation and overcome resistance mechanisms. Nanotechnology offers a promising solution by enabling the development of nanomaterials that can address these limitations.

Nanomaterials, including nanoparticles (NPs), nanogels, liposomes, and micelles, can be engineered for precise drug delivery (14, 15). By utilizing the enhanced permeability and retention (EPR) effect, these materials can deliver therapeutic agents like ICIs, tumor antigens, and chemotherapeutic drugs directly to the tumor site (16, 17). Additionally, nanomaterials can modulate the immune response by mimicking pathogen-associated molecular patterns (PAMPs), thereby triggering innate immune responses and enhancing antigen presentation (18, 19). NP-based vaccines, which co-deliver tumor antigens and immune-stimulatory agents, have demonstrated potential in amplifying the immune system’s ability to target and eliminate melanoma cells (20). The integration of nanotechnology into melanoma immunotherapy also presents a solution to the unique challenges of the TME. Nanomaterials can deliver melanoma-specific antigens to dendritic cells (DCs), activating CD8^+^ T cells and boosting targeted anti-tumor responses (21, 22). The combination of NPs with ICIs has shown promise in counteracting the immunosuppressive effects of the TME, enhancing therapeutic efficacy (23). Furthermore, NPs can be tailored to suppress the activity of immunosuppressive cells like regulatory T cells (Tregs) and myeloid-derived suppressor cells (MDSCs), while simultaneously improving T cell function (22).

Nanomaterials offer a transformative approach to melanoma immunotherapy, enabling more precise treatment, modulation of the immune microenvironment, and overcoming the limitations of conventional therapies (24–26). As research progresses, nanotechnology is expected to play a growing role in the development of personalized and effective melanoma treatments, improving patient outcomes and reshaping the therapeutic paradigm for this challenging cancer.

## 2 Molecular profiling, targeted therapies and response to ICB of melanoma

### 2.1 Molecular profiling

The pathophysiology of melanoma is predominantly driven by genetic mutations, many of which are caused by DNA damage resulting from UV radiation exposure (11). These mutations primarily affect key genes involved in regulating cell growth, survival, and apoptosis. Notably, mutations in the BRAF, NRAS, and p53 genes are commonly observed in melanoma, driving unregulated cell proliferation, resistance to programmed cell death, and the eventual development of malignant tumors (27–31). The BRAF V600E mutation, in particular, is one of the most frequently identified genetic alterations in melanoma and is a key target for targeted therapies.

**Figure 1:**
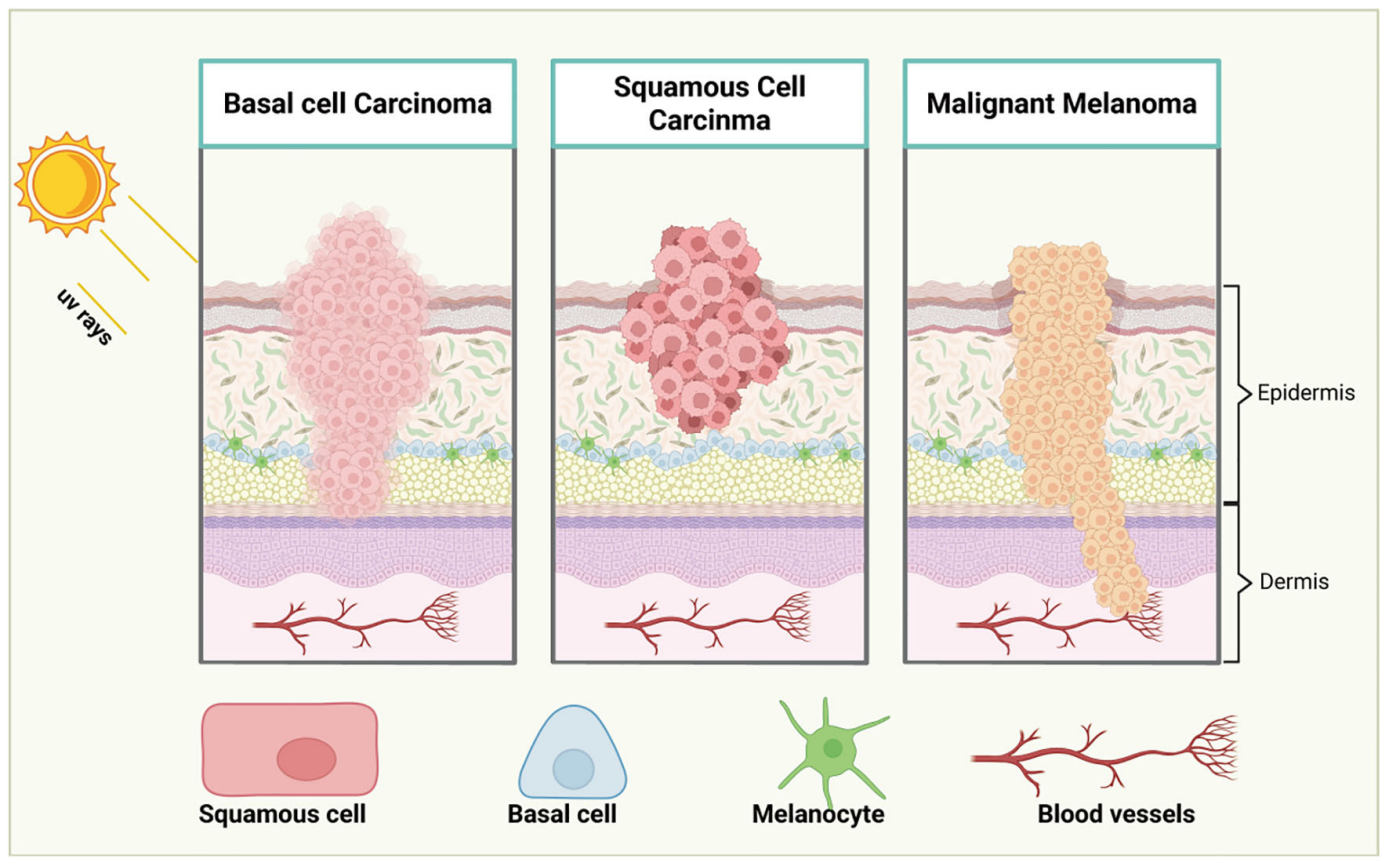
Stages of melanoma progression. The illustration delineates the progression of melanoma through distinct stages: from initial to advanced phases. In Stage I, melanoma is confined to the epidermis, originating from melanocytes and is typically small and localized. In Stage II, the melanoma grows in size and thickness, yet remains within the epidermis. By Stage III, the melanoma breaches its confinement, spreading into regional lymph nodes and adjacent soft tissues, as malignant cells begin their migration via lymphatic vessels. Stage IV marks the dissemination of melanoma to distant lymph nodes, tissues, and organs far from the primary tumor site. Cancer cells may also travel through the bloodstream to distant organs such as the lungs, liver, or brain. This progression underscores the critical importance of early detection for improved patient prognosis. *Created with BioRender*.

Early-stage melanoma often manifests as a new or changing nevus (mole), characterized by irregular borders, asymmetry, and a range of color variations, including brown, black, blue, or even white or red. While these early lesions may initially appear benign, they are often precursors to malignant melanoma if left unchecked (32). In its early stages, melanoma may be asymptomatic, which can make detection challenging. However, as melanoma progresses, patients may experience symptoms such as itching, tenderness, or bleeding, which serve as red flags for potential malignancy. As melanoma advances, it invades deeper layers of the skin, allowing cancer cells to penetrate the bloodstream and lymphatic system, facilitating metastasis to distant organs. This metastatic ability is a defining feature of melanoma’s aggressive nature and highlights the critical importance of early detection and intervention (33). Untreated melanoma has a high potential for rapid progression, leading to poor survival outcomes. Early molecular profiling and genetic testing can aid in the identification of specific mutations, allowing for more accurate diagnoses and better-targeted treatment strategies. The stages of melanoma progression was showed in **Figure 1**.

### 2.2 Targeted therapies

**Figure 2:**
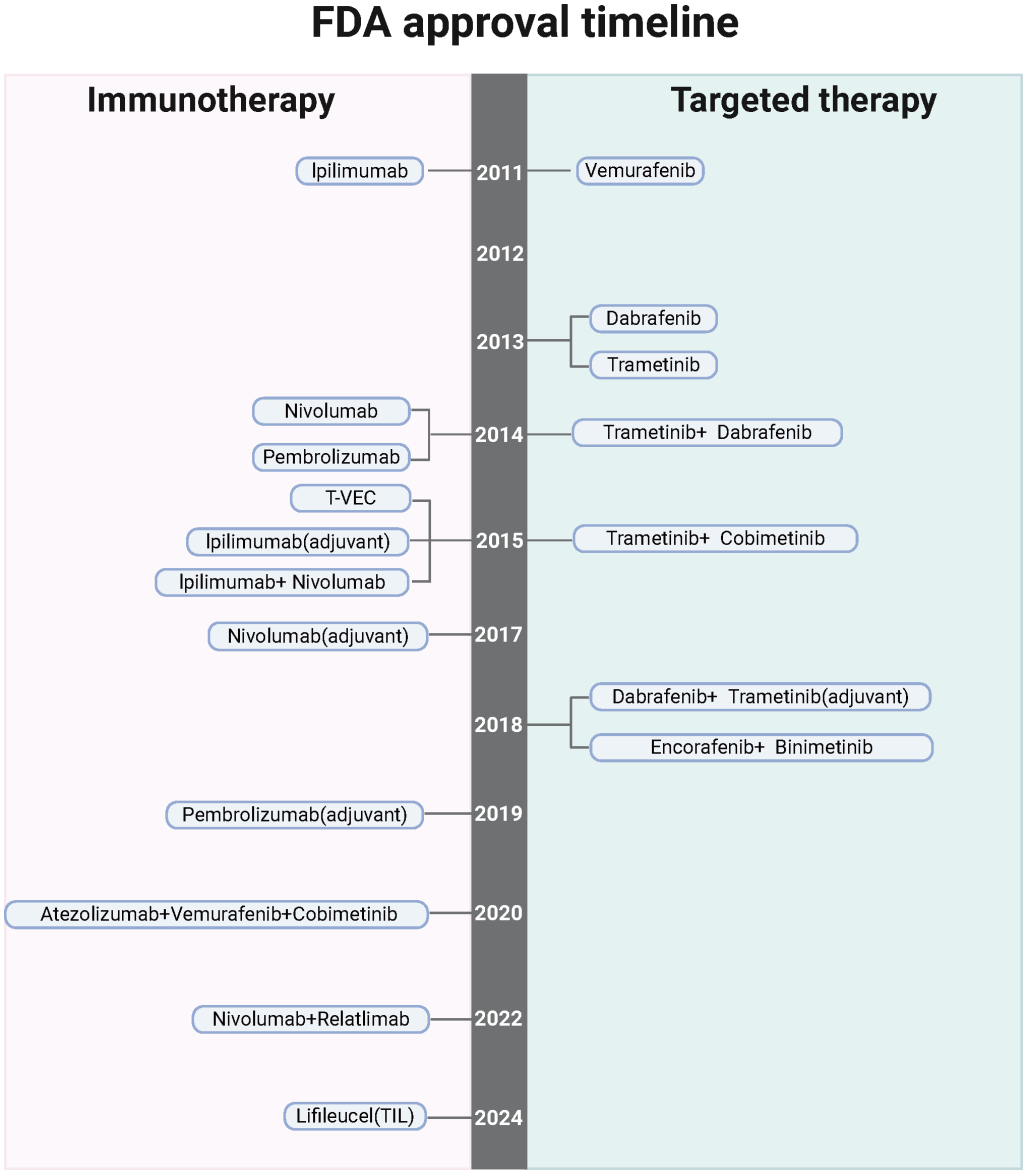
The timeline highlights FDA-sanctioned targeted treatments and immunotherapies for melanoma, underscoring the potential, barriers, and hurdles associated with nano-immunotherapy in this context. *Created with BioRender*.

Molecular evaluation of driver mutations has become standard practice in guiding treatment decisions for melanoma patients, particularly those undergoing systemic therapy (**Figure 2**). Among the most commonly observed mutations is in the BRAF oncogene, found in approximately 50% of melanoma cases. The discovery of BRAF mutations catalyzed the development of targeted therapies, significantly improving patient outcomes (34, 35). BRAF is a serine-threonine kinase within the RAS-RAF-MEK-ERK signaling pathway. Mutations such as BRAF V600E lead to the constitutive activation of MEK and ERK, driving uncontrolled tumor cell growth (36, 37). Other mutations commonly found in melanoma include NRAS (28% of cases), NF1 (14%), and KIT (15-20% in acral and mucosal melanomas) (38–43). These mutations can be detected through molecular testing of primary tumor samples, metastatic tissues, or regional lymph nodes.

The approval of the BRAF inhibitor vemurafenib marked a significant advancement in the treatment of metastatic melanoma harboring BRAF V600 mutations (44). Clinical studies have shown that vemurafenib induces partial or complete tumor regression in patients with BRAF V600E-mutated melanoma (45). Similarly, MEK inhibitors like trametinib have shown considerable improvements in progression-free survival (PFS) and overall survival (OS) when combined with BRAF inhibitors, representing a paradigm shift in melanoma treatment (37, 46). The combination of BRAF and MEK inhibitors, such as dabrafenib and trametinib, has proven particularly effective, enhancing PFS and OS while minimizing toxicity compared to BRAF inhibition alone (45). These combination regimens are now the standard for treating BRAF-mutant metastatic melanoma.

In addition to their use in metastatic melanoma, BRAF and MEK inhibitors have also been explored in the adjuvant setting. The COMBI-AD phase III trial demonstrated that patients with resected stage III BRAF-mutated melanoma who received dabrafenib and trametinib as adjuvant therapy had longer relapse-free survival (RFS) and distant metastasis-free survival compared to those receiving placebo (47). This led to the FDA approval of the regimen in 2018. While combination BRAF-MEK inhibition has become a cornerstone in treating BRAF-mutant melanoma, ongoing research is focused on its optimal role in the adjuvant setting, particularly in comparison with adjuvant immunotherapy. Moreover, there is emerging interest in the use of neoadjuvant BRAF and MEK inhibitors to improve treatment outcomes for melanoma patients.

For melanomas with mutations outside of BRAF, targeted therapies offer promise, especially in second-line settings. Approximately 15-20% of acral and mucosal melanomas harbor activating mutations in the KIT gene (42, 48). In such cases, the tyrosine kinase inhibitor imatinib has been shown to extend PFS, with a disease control rate of around 55%. Patients with cutaneous melanoma harboring rare TRK gene fusions may benefit from therapies like larotrectinib and entrectinib, designed specifically to target these fusions (49, 50). Additionally, mutations in NRAS are present in 15-20% of BRAF-wildtype cutaneous melanomas (41). For these patients, first-line treatment with immunotherapy is typically recommended; however, if progression occurs, the addition of the MEK inhibitor binimetinib or participation in clinical trials can provide further therapeutic options.

### 2.3 Response to immune checkpoint blockade

Nano-immunotherapy has emerged as a pivotal approach in melanoma treatment, primarily by activating immune cells. This innovative strategy has shown promise, yet faces numerous challenges and obstacles. The TME significantly impacts melanoma’s transcriptomic landscape, meaning nano-immunotherapy not only induces tumor cell death but may also drive shifts in melanoma cell states and meta-programs (51). Clinical melanoma samples analyzed through bulk RNA sequencing before and during immunotherapy have revealed that immune-related genes are upregulated in patients who respond to treatment, whereas mesenchymal (MES)-related genes are predominant in non-responders (52–54). However, these analyses are complicated by overlapping gene signatures among melanoma cell states/meta-programs and various TME components such as immune cells and cancer-associated fibroblasts (CAFs) (55–57).

Single-cell RNA sequencing has identified the first cancer cell-specific meta-program linked to T cell exclusion and resistance to immunotherapy, characterized by increased levels of CDK4, MYC, and CDK7 (58–60). A recent comparative study examining biopsies before treatment and during early treatment found a rare cell population enriched with a MES transcriptional program driven by the transcription factor TCF4 (ITF2), notably present in early treatment samples of non-responders (61). Although lineage tracing or barcoding would be needed to confirm this, it’s plausible that ICB-induced transcriptional reprogramming leads to an increase in MES cells. This hypothesis is supported by earlier studies demonstrating that adoptive T cell transfer can induce melanoma cell de-differentiation in preclinical models.

Genetic targeting of TCF4 or using bromodomain and extra-terminal motif (BET) inhibitors has been shown to enhance melanoma cell recognition by T cells in co-culture experiments, increasing the sensitivity of melanoma lesions to ICB in mouse models (62–64). These findings indicate that MES melanoma cells are particularly adept at evading immune responses, possibly by downregulating antigen processing and presentation pathways and the T cell-attracting chemokine CXCL10 (65, 66). Their increased migratory ability may further contribute to immune evasion. However, the mechanism by which this small MES cell population promotes ICB resistance within the tumor ecosystem remains unclear. One hypothesis is that they may exert influence in a paracrine, or non-cell-autonomous manner, perpetuating the cancer immune cycle and reinforcing antitumor immune response as immune-stimulatory factors accumulate. Conversely, responders to ICB therapies exhibit increased antigen-presenting cell populations, aligning with studies that associate MHC class II (HLA-DR) molecule expression in melanomas with higher densities of CD8^+^ tumor-infiltrating lymphocytes (TILs) and favorable anti-PD-1 therapy responses (67, 68). These insights have deepened our understanding of melanoma ecosystem evolution under ICB treatment and suggested strategies to convert non-responding lesions into responding ones by modulating the gene regulatory network in de-differentiated melanoma MES cells.

**Figure 3:**
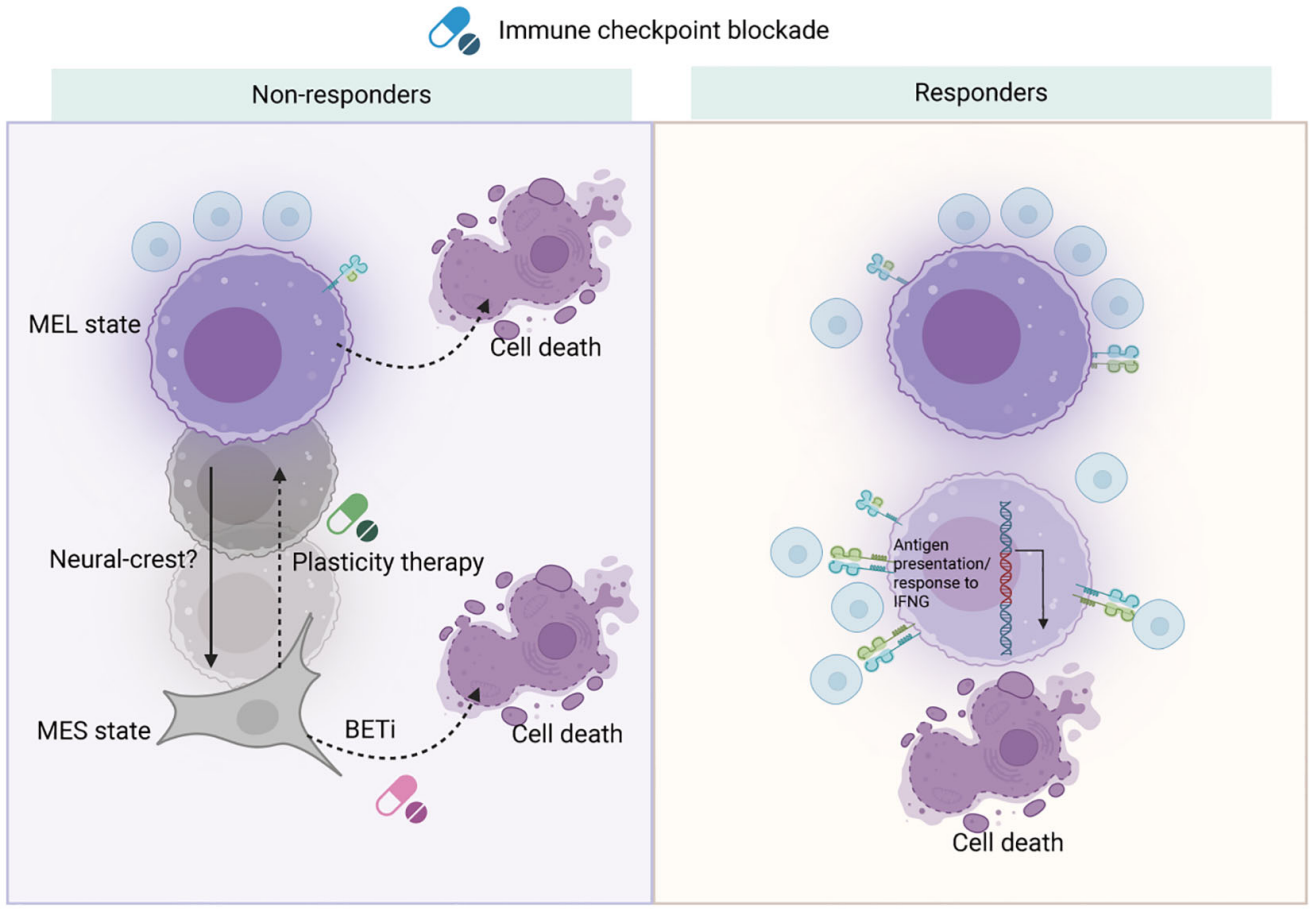
Cell states and response to ICB of melanoma. Nano-immunotherapy presents new avenues for addressing melanoma by utilizing the body’s immune system. ICB can trigger reprogramming of melanoma cell states and meta-programs via immune cell activation. However, in non-responsive patients, melanocytic melanoma cells may escape recognition and destruction by undergoing cytokine-induced de-differentiation. To counteract resistance to ICB, one strategy is to target these de-differentiated melanoma cells with agents like bromodomain and extra-terminal motif (BET) inhibitors. Alternatively, a ‘plasticity therapy’ could be designed to either inhibit de-differentiation or encourage differentiation. In patients who respond well to treatment, activated immune cells can effectively identify melanocytic melanoma cells, which can be tracked by a strong induction of the antigen presentation meta-program. *Created with BioRender*.

Overall, cell states and response to ICB of melanoma was showed in **Figure 3**. Future strategies might focus on pharmaceutical interventions that limit melanoma cell plasticity, preventing the emergence of MES cells or directing them towards a more differentiated state, thereby enhancing ICB response by mitigating immune evasion. Approaches targeting cell plasticity and the MES-to-MEL differentiation trajectory should be considered in forthcoming melanoma therapies to improve outcomes for ICB-resistant patients.

Despite significant advances, successful immunotherapy in melanoma faces several mechanistic challenges. Tumor-induced immunosuppression, heterogeneous tumor microenvironments, and inefficient delivery of therapeutic agents often limit response rates and durability of treatment. Conventional approaches frequently struggle to achieve targeted and controlled modulation of immune pathways due to biological barriers, off-target effects, and the dynamic nature of tumor-immune interactions. Nanoparticles offer a versatile platform to surmount these obstacles by enabling precise delivery of immune modulators directly to tumor sites, thereby enhancing local efficacy while reducing systemic toxicity. Their capacity for surface modification allows for targeted engagement with specific immune cells or tumor components, fostering reprogramming of the tumor microenvironment (TME) toward an immunostimulatory state. Furthermore, nanomaterials can co-deliver multiple therapeutic agents, synchronize immune activation with tumor ablation, and respond adaptively to the tumor milieu through stimuli-responsive designs. Collectively, these features position nanoparticles as powerful tools to mechanistically overcome the barriers limiting current immunotherapies and to realize more personalized, durable, and effective melanoma treatments.

## 3 The mechanism of NP-based drug delivery systems

NP-based drug delivery systems have emerged as a promising strategy for targeted and controlled drug delivery, offering substantial improvements over conventional drug delivery methods (69–71). These NPs can be internalized by cells via various mechanisms, each with distinct advantages tailored to specific therapeutic applications (72–74). The most commonly observed mechanisms of internalization include receptor-mediated endocytosis, phagocytosis, and pinocytosis (75, 76).

Receptor-mediated endocytosis is a highly selective process where NPs are internalized by cells through specific interactions between ligands on the NP surface and corresponding receptors on the cell membrane (77). This binding triggers the formation of clathrin-coated pits, which invaginate to form vesicles containing the NPs. This mechanism allows for precise drug delivery, as it ensures targeting to specific cell types or tissues, making it particularly effective for precision therapies. In contrast, phagocytosis, primarily carried out by specialized immune cells such as macrophages and DCs, involves the engulfment of larger particles, including NPs (78). The particles are enclosed by pseudopodia, forming phagosomes that fuse with lysosomes for degradation. This pathway is particularly beneficial for delivering therapeutic agents to immune cells or for immune-modulatory therapies. Pinocytosis, often referred to as “cell drinking,” involves the non-selective uptake of extracellular fluids and small molecules. This process occurs via two primary pathways: clathrin-mediated endocytosis, where NPs are engulfed by clathrin-coated pits, and caveolae-mediated endocytosis, which involves small membrane invaginations called caveolae that facilitate NP internalization (79).

To enhance the cellular uptake of NPs, several surface modification strategies have been developed, aimed at optimizing drug delivery efficiency (80). One of the most common strategies is ligand conjugation, where specific ligands such as antibodies, peptides, or aptamers are attached to the NP surface (81). These ligands selectively bind to cell surface receptors, promoting receptor-mediated endocytosis and improving targeted drug delivery. Another method involves modifying the surface charge of NPs, which can improve interactions with the negatively charged cell membranes, thereby enhancing internalization (82). Furthermore, the use of cell-penetrating peptides (CPPs) to coat NPs can facilitate their traversal across the cell membrane, promoting efficient intracellular drug delivery (83, 84).

The controlled release of the drug cargo from NPs is another critical factor in the design of effective drug delivery systems (85, 86). Parameters such as NP size, shape, and composition significantly influence the rate at which the drug is released. Smaller NPs generally release their cargo more rapidly due to their larger surface area-to-volume ratio, which enhances diffusion (87). The shape of the NPs also impacts release rates, with rod-shaped or porous structures often enabling more controlled release through altered internal configurations (88). Biodegradable NPs offer the added advantage of gradually releasing their drug payload as they degrade, allowing for a more controlled release profile (89). On the other hand, non-biodegradable NPs may require external stimuli, such as temperature, pH changes, or enzyme activity, to trigger the release of their drug cargo (90). This has led to the development of stimuli-responsive NPs, which can release drugs in response to specific conditions in the TME, such as the acidic pH or the presence of tumor-associated enzymes (91).

**Figure 4:**
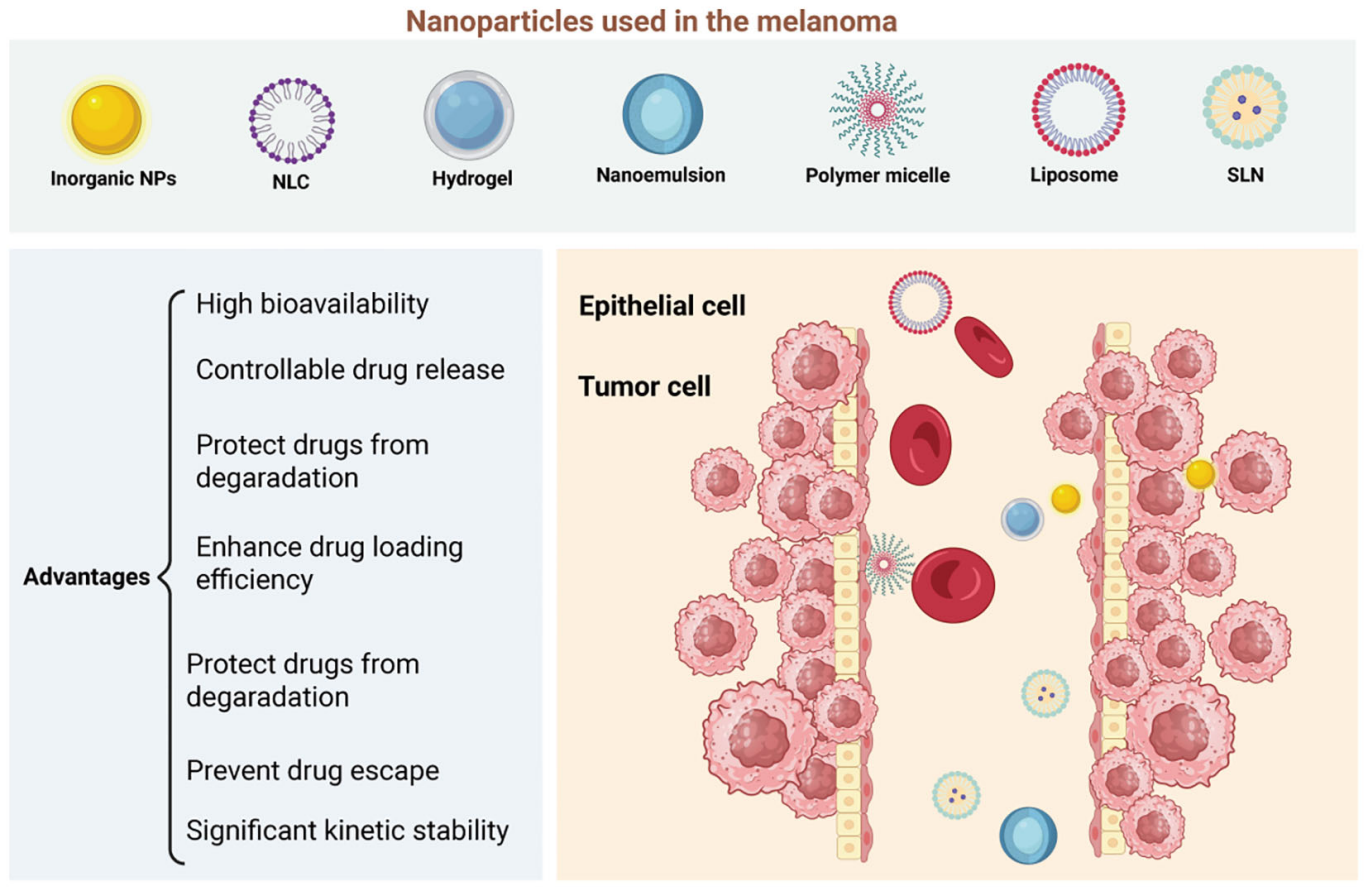
Nanomaterials as platforms for nano-drug delivery systems (nano-ddss) in melanoma. Nanomaterials such as liposomes, nanostructured lipid carriers (NLCs), solid lipid nanoparticles (SLNs), hydrogels, nanoemulsions, polymer micelles, and inorganic nanoparticles (NPs) are promising platforms for nano-drug delivery systems (nano-DDSs) in melanoma treatment, offering several advantages. Liposomes and lipid-based nanoparticles (NLCs, SLNs) improve drug solubility, bioavailability, and stability while reducing toxicity. NLCs, in particular, allow for sustained release and can encapsulate a variety of hydrophobic drugs. Hydrogels provide localized, stimulus-responsive drug delivery, while nanoemulsions enhance the solubility and skin penetration of poorly soluble drugs. Polymer micelles improve drug stability and release by responding to changes in the tumor microenvironment. Inorganic nanoparticles, like gold and silica, are versatile carriers that enable targeted delivery and theranostic applications. These nanomaterials’ ability to enhance drug delivery, stability, and specificity makes them promising candidates for improving melanoma therapies. *Created with BioRender*.

NP-based drug delivery systems hold significant promise for improving drug targeting and controlled release. By optimizing surface modifications to enhance cellular uptake and designing NPs that respond to specific environmental cues for drug release (80, 92), it is possible to create more effective and precise therapies for a wide range of diseases, including melanoma (**Figure 4**). A deeper understanding of the mechanisms of NP internalization and drug release will be crucial to advancing the clinical applications of NP-based therapies, ensuring better therapeutic outcomes and minimizing side effects.

## 4 Nano−based therapeutics for immunotherapy in melanoma

### 4.1 Lipid−based NPs

LNPs have emerged as versatile platforms for overcoming key challenges in melanoma immunotherapy, including TME immunosuppression, limited antigen delivery efficiency, and systemic toxicity (**Table 1**). Their unique structural adaptability enables precise modulation of immune responses through multifunctional designs.

**Table 1:** Lipid based nanoparticles for immunotherapy in melanoma.

Nanosystem	Approaches	Biological function	Reference
Lipid-calcium-phosphate (LCP) nanoparticles	Co-delivery of TGF-β siRNA and LCP vaccines	Overcomes TME immunosuppression, enhances antigen presentation, and improves antitumor responses in advanced melanoma	(93)
Lipid-polyarginine-hyaluronic acid (LPH) nanoparticles	Co-delivery of TGF-β siRNA and LCP vaccines	Silences immunosuppressive signals, enhances antigen presentation	(93)
Zinc-chelating lipid-coated zinc phosphate hybrid nanoparticles (LZnP NPs)	High peptide loading efficiency and MHC class I/II presentation	Promotes cross-presentation via MHC class I/II, enhances immune responses across diverse HLA haplotypes	(94)
Immunogenic nanoliposomes with TLR4 agonist KDO2	Encapsulation of hexavalent melanoma vaccine and TLR4 agonist	Enhances CD8+/CD4+ T cell priming, synergizes with checkpoint inhibitors, prolongs survival	(95)
IL-13-functionalized long-circulating liposomes (IL-13-LCL-SIM) and PEGylated extracellular vesicles (PEG-EV-DOX)	Disrupts TAM-melanoma cell crosstalk, delivers doxorubicin	Selectively depletes protumoral M2-TAMs, induces oxidative stress and apoptosis, reshapes TME, suppresses metastasis	(96)
Transdermal LPPC-DOX-CpG nanocomplex	Combines cationic liposomes, doxorubicin, and CpG ODNs	Activates APCs and CTLs, inhibits primary and metastatic lesions	(97)
pLCGM-OVA nanoagonist	Co-delivery of Cu/Mn ions and ovalbumin antigen	Induces ROS-mediated cuproptosis, activates cGAS-STING pathway, amplifies DC maturation and CTL infiltration, theranostic application	(98)

#### 4.1.1 Overcoming TME immunosuppression

In advanced melanoma, the immunosuppressive TME dominated by TGF-β severely limits therapeutic efficacy. While lipid-calcium-phosphate (LCP) NPs showed early promise in antigen delivery for primary melanoma, their efficacy in late-stage disease was constrained by TGF-β-mediated immune suppression (93). To address this, lipid-polyarginine-hyaluronic (LPH) acid NPs were engineered to co-deliver TGF-β siRNA and LCP vaccines. This dual-action strategy silenced immunosuppressive signals while enhancing antigen presentation, significantly improving antitumor responses in advanced melanoma models (93).

#### 4.1.2 Enhancing antigen delivery and immunogenicity

Antigen encapsulation efficiency and MHC-restricted immune activation are critical for vaccine efficacy. Zinc-chelating lipid-coated zinc phosphate hybrid NPs (LZnP NPs) leverage zinc’s coordination properties to achieve >90% peptide loading efficiency. Their lipid bilayer enhances colloidal stability and biocompatibility, while the hybrid core promotes cross-presentation via MHC class I/II pathways, broadening T cell responses across diverse HLA haplotypes (94). Building on this, a hexavalent melanoma vaccine encapsulated in immunogenic nanoliposomes with TLR4 agonist KDO2 demonstrated enhanced CD8^+^/CD4^+^ T cell priming. This design overcame peptide instability and drug resistance, synergizing with checkpoint inhibitors to prolong survival (95).

#### 4.1.3 Combinatorial strategies for dual targeting

Lipid-based systems enable spatiotemporal control of chemoimmunotherapy. For example, IL-13-functionalized long-circulating liposomes (IL-13-LCL-SIM) and PEGylated extracellular vesicles (PEG-EV-DOX) were designed to disrupt crosstalk between tumor-associated macrophages (TAMs) and melanoma cells. IL-13-LCL-SIM selectively depleted protumoral M2-TAMs, while PEG-EV-DOX delivered doxorubicin (DOX) to tumor cells, inducing oxidative stress and apoptosis. This dual targeting reshaped the TME and suppressed metastatic progression (96). Similarly, a transdermal LPPC-DOX-CpG nanocomplex combined cationic liposomes (LPPC), DOX, and CpG ODNs. This system activated antigen-presenting cells (APCs) and cytotoxic T lymphocytes (CTLs), inhibiting both primary and metastatic lesions in visceral organs (97).

#### 4.1.4 Activating novel immunogenic pathways

Recent advances integrate lipid NPs with immunogenic cell death (ICD) inducers. The pLCGM-OVA nanoagonist co-delivers Cu/Mn ions and ovalbumin antigen, responding to acidic TME for controlled release. Cu ions induce ROS-mediated cuproptosis, while Mn ions activate the cGAS-STING pathway, amplifying DC maturation and CTL infiltration. Remarkably, pLCGM-OVA’s intrinsic MRI T1 contrast enabled real-time monitoring of therapeutic responses, exemplifying a theranostic paradigm (98).

### 4.2 Polymeric nanoparticles

PNPs have emerged as a transformative platform in melanoma immunotherapy, leveraging their structural versatility to enhance immune activation, optimize drug delivery, and reprogram the TME (**Table 2**). These systems integrate multifunctional designs to address the complex immunosuppressive landscape of melanoma.

**Table 2:** Polymeric nanoparticles for immunotherapy in melanoma.

Nanosystem	Approaches	Biological function	Reference
Tobacco mosaic virus (TMV)-based PNPs	Co-loaded with TLR-7 agonist 1V209 and photothermal polydopamine (PDA)	Synergistic photothermal-immunotherapy; induces tumor-specific CD8+ T cells and prolonged survival in melanoma	(99)
Nano-hydroxyapatite (nHA) and GM-CSF hydrogel	Thermosensitive hydrogel combining nHA and GM-CSF	Dual immunomodulation: nHA induces tumor cell apoptosis, GM-CSF enhances DC recruitment and antigen presentation	(100)
Peptide nanotube (PNT)-based PNPs	Delivery of cyclic dinucleotide c-di-GMP for STING pathway activation	Enhances cytosolic STING activation, boosts type I interferon production, CD8+ T cell-mediated tumor suppression	(101)
Bioengineered nanogels (CDNPs)	Co-encapsulation of BRAF and COX2 inhibitors, combined with anti-PD-1	Induces melanoma cell pyroptosis, enhances cytotoxic T lymphocyte infiltration and tumor regression	(102)
Ato/cabo@PEG-TK-PLGA system	Dual transdermal and intravenous delivery, targets MDSCs via H2O2-triggered release	Suppresses MDSC-mediated T cell inhibition, synergizes with PDT to eradicate primary tumors and prevent metastasis	(103)
Fluorocarbon-modified chitosan (FCS) nanoparticles	Loaded with poly(I:C), paired with anti-PD-1 therapy	Reprograms the TME, activates DCs, enhances CD8+ T cell priming, improves checkpoint inhibitor efficacy	(104)
Poly-metformin-based carboxymethyl chitosan (PolyMetCMCS) nanogels	Selectively promotes M1 macrophage polarization	Activates DC maturation, recruits cytotoxic T cells, inhibits tumor growth, establishes long-term immunological memory	(105)

#### 4.2.1 Immune activation via PAMP engagement

PNPs excel in delivering immune-stimulating payloads to activate innate and adaptive immunity. For instance, tobacco mosaic virus (TMV)-based PNPs co-loaded with the TLR-7 agonist 1V209 and photothermal polydopamine (PDA) achieved synergistic photothermal-immunotherapy. Under laser irradiation, the 1V209-TMV-PDA system induced localized hyperthermia for tumor ablation while triggering systemic antitumor immunity, marked by elevated tumor-specific CD8^+^ T cells and prolonged survival in melanoma-bearing mice (99). Complementing this, a thermosensitive hydrogel combining nano-hydroxyapatite (nHA) and GM-CSF demonstrated dual immunomodulation: nHA selectively induced tumor cell apoptosis via calcium overload, while GM-CSF amplified DC recruitment and antigen presentation. This combination outperformed monotherapies, highlighting the potential of PNPs to orchestrate spatially controlled immune activation (100).

#### 4.2.3 Precision delivery of STING agonists and combinatorial therapeutics

The cyclic dinucleotide c-di-GMP, a potent STING pathway activator, was effectively delivered using peptide nanotube (PNT)-based PNPs. The c-di-GMP-PNT complex enhanced cytosolic STING activation in APCs, resulting in robust type I interferon production and CD8^+^ T cell-mediated tumor suppression. Notably, this strategy not only controlled primary melanoma but also inhibited metastatic spread, underscoring its systemic immunomodulatory capacity (101). Further advancing combination therapy, bioengineered nanogels (CDNPs) co-encapsulating BRAF and COX2 inhibitors induced melanoma cell pyroptosis—a highly ICD mode. When combined with anti-PD-1 antibodies, CDNPs synergistically enhanced CTL infiltration and tumor regression, providing a blueprint for overcoming ICB resistance (102).

#### 4.2.4 Reprogramming immunosuppressive TME

PNPs are uniquely suited to dismantle immunosuppressive networks within the TME. The Ato/cabo@PEG-TK-PLGA system, administered via dual transdermal and intravenous routes, targeted MDSCs through localized hydrogen peroxide (H2O2)-triggered release of atovaquone and cabozantinib. This approach suppressed MDSC-mediated T cell inhibition while synergizing with photodynamic therapy (PDT) to eradicate primary tumors and prevent metastasis (103). Similarly, fluorocarbon-modified chitosan (FCS) NPs loaded with poly(I:C) reprogrammed the TME by activating DCs and enhancing CD8^+^ T cell priming. When paired with anti-PD-1 therapy, this system significantly improved checkpoint inhibitor efficacy, demonstrating PNPs’ ability to convert “cold” tumors into immunogenic niches (104).

#### 4.2.5 Macrophage polarization and postoperative immunity

A breakthrough in macrophage-centric therapy was achieved using poly-metformin-based carboxymethyl chitosan (PolyMetCMCS) nanogels. These PNPs selectively promoted M1 macrophage polarization via STAT3/NF-κB pathway modulation, which in turn activated DC maturation and cytotoxic T cell recruitment. In postsurgical melanoma models, PolyMetCMCS nanogels not only inhibited residual tumor growth but also established long-term immunological memory, preventing recurrence—a critical advancement for adjuvant immunotherapy (105).

### 4.3 Protein-based NPs

Protein-based NPs (PNPs) have emerged as a transformative strategy to counteract melanoma’s immunosuppressive TME, addressing key challenges such as immune evasion, defective antigen presentation, and impaired cytotoxic T cell activity (**Table 3**). By leveraging the inherent biocompatibility and molecular specificity of proteins, these nanoplatforms enable precise modulation of immune pathways, offering novel solutions to reinvigorate antitumor immunity.

**Table 3:** Protein based nanoparticles for immunotherapy in melanoma.

Nanosystem	Approaches	Biological function	Reference
Trimeric trap proteins	Sequester Wnt5a, delivered via PNPs	Neutralizes Wnt5a’s pro-fibrotic effects, restores DC functionality, enhances CTL infiltration, improves survival	(106)
Lipid-protein hybrid nanoparticles (LPNs)	Delivers plasmid DNA encoding Fas (hCOFAS01)	Restores Fas expression, sensitizes melanoma to CTL attack, enhances tumor cell apoptosis, reprograms TME	(107)
Cyclic peptide-PNP system	Delivers cyclic peptides to block CD47-SIRPα interactions	Disrupts immune evasion, promotes macrophage phagocytosis of tumor cells, enhances innate immunity	(108)

#### 4.3.1 Disrupting tumor-derived immunosuppression: Targeting Wnt5a in BRAF-mutant melanoma

In BRAF-mutant melanomas, Wnt5a overexpression drives stromal fibrosis and DC tolerization, creating an immune-excluded TME. To reverse this immunosuppressive axis, trimeric trap proteins were engineered to sequester Wnt5a and delivered via PNPs. This approach neutralized Wnt5a’s pro-fibrotic effects, restoring DC functionality and enabling CTL infiltration. When combined with low-dose DOX—a chemotherapy agent that induces ICD—the trapped Wnt5a-PNP system synergistically enhanced tumor antigen release and cross-presentation. Preclinical studies demonstrated that this dual-action strategy not only suppressed primary tumor growth but also established systemic immune memory, significantly improving survival in aggressive melanoma models (106).

#### 4.3.2 Resurrecting apoptotic signaling: Restoring Fas-mediated immune surveillance

Epigenetic silencing of the Fas death receptor in melanoma cells allows them to evade CTL-mediated apoptosis. To overcome this resistance, lipid-protein hybrid NPs (LPNs) were designed to deliver plasmid DNA encoding Fas (hCOFAS01). These PNPs efficiently transfected tumor cells, reinstating Fas expression and sensitizing melanoma to CTL attack. The restored Fas signaling cascade triggered tumor cell apoptosis while concurrently reprogramming the TME, as evidenced by increased CTL infiltration and reduced immunosuppressive cytokine levels. This gene-editing strategy highlights PNPs’ capacity to resurrect defective apoptotic pathways and reverse immune tolerance, providing a blueprint for restoring tumor cell susceptibility to immune elimination (107).

#### 4.3.3 Unleashing innate immunity: blocking CD47-SIRPα “don’t eat me” signals

The CD47-SIRPα axis is a master regulator of macrophage-mediated immune evasion in melanoma. To disrupt this checkpoint, cyclic peptides mimicking the SIRPα-binding domain of CD47 were synthesized and delivered via PNPs. Engineered with a threonine-to-phenylalanine mutation (T/F variant), these cyclic peptides exhibited enhanced binding affinity for human SIRPα compared to linear counterparts. In human melanoma models, the peptide-PNP system competitively inhibited CD47-SIRPα interactions, thereby unleashing macrophage phagocytosis of tumor cells. Notably, the species-specific efficacy of these peptides—demonstrating higher activity in human cells than in murine systems—underscores their translational potential for clinical immunotherapy. This work exemplifies how PNPs can be tailored to block immune checkpoints and reactivate innate immune effector functions (108).

### 4.4 Metallic-based NPs

MNPs are revolutionizing melanoma immunotherapy by exploiting their intrinsic catalytic, magnetic, and optical properties to orchestrate multimodal antitumor immune responses (**Table 4**). These nanoplatforms address the immunosuppressive TME through mechanisms spanning macrophage polarization, ICD, and vascular remodeling, offering unprecedented opportunities for synergistic therapy.

**Table 4:** Metallic based nanoparticles for immunotherapy in melanoma.

Nanosystem	Approaches	Biological function	Reference
Iron oxide nanoparticles (FMT-pIC)	Functionalized with TLR3 agonist poly(I:C) to polarize M2-TAMs into M1 phenotypes	Enhances phagocytosis, cytokine secretion (e.g., TNF-α, IL-12), activates CTLs, suppresses melanoma growth	(109, 110)
Manganese-zinc sulfide (ZMS) nanoparticles	Co-loaded with IR780 for chemodynamic therapy (CDT) and photothermal therapy (PTT)	Generates ROS, induces hyperthermia, activates DCs, promotes CTL infiltration, amplifies cGAS-STING pathway	(111)
Redox-responsive magnetic nanocarriers (rMMNs)	Glutathione-triggered release of miR-30a-5p, polarizes TAMs to M1 states, inhibits angiogenesis	Silences E2F7, enhances IL-6/IL-12 secretion, recruits CTLs, disrupts angiogenesis	(112, 113)
Metal-organic frameworks (MOF) TPL@TFBF	Releases Fe^3+^ and tannic acid to induce ferroptosis and pyroptosis in melanoma cells	Triggers immunogenic cell death, recruits DCs, enhances CD8+ T cell priming, enhances anti-PD-1 therapy efficacy	(114)

#### 4.4.1 Macrophage-centric immunotherapy via iron oxide NPs

Iron oxide NPs, such as ferumoxytol (FMT), have emerged as potent tools for reprogramming TAMs. Functionalization of FMT with TLR3 agonist poly(I:C) (FMT-pIC) converted immunosuppressive M2-TAMs into tumoricidal M1 phenotypes, enhancing phagocytosis and pro-inflammatory cytokine secretion. This polarization disrupted stromal barriers and activated CTLs, significantly suppressing melanoma growth and metastasis. The therapeutic potential of iron oxides was further amplified in a nanovaccine formulation combining FMT with TLR4 agonist MPLA, which synergized with α-CD40 antibodies to induce tumor necrosis and systemic immunity in checkpoint inhibitor-resistant models (109).

#### 4.4.2 Combinatorial chemodynamic-photothermal activation

Manganese-zinc sulfide (ZMS) NPs co-loaded with near-infrared dye IR780 exemplify the integration of chemodynamic therapy (CDT) and photothermal therapy (PTT). Under laser irradiation, ZMS-IR780 generated cytotoxic reactive oxygen species (ROS) via Fenton-like reactions while inducing localized hyperthermia. This dual action triggered ICD, releasing damage-associated molecular patterns (DAMPs) that activated DCs and promoted CTL infiltration. Concurrently, manganese ions released from ZMS activated the cGAS-STING pathway, amplifying type I interferon production and establishing durable antitumor immunity against both primary and metastatic lesions (110).

#### 4.4.3 Redox-responsive gene delivery and angiogenesis inhibition

Redox-responsive magnetic nanocarriers (rMMNs) addressed ocular melanoma’s therapeutic resistance through glutathione-triggered release of miR-30a-5p, which silenced the oncogenic transcription factor E2F7 to suppress tumor proliferation. Simultaneously, iron ions within rMMNs polarized TAMs to M1 states, enhancing IL-6/IL-12 secretion and recruiting CTLs. In parallel studies, FMT-pIC NPs inhibited melanoma angiogenesis by downregulating VEGF and angiopoietin-2, disrupting endothelial cell networks and synergizing with macrophage-driven immune activation to create a hostile TME for tumor survival (111).

#### 4.4.4 Orchestrating ICD via metal-organic frameworks (MOFs)

The folate-modified MOF NP TPL@TFBF demonstrated precision induction of ferroptosis and pyroptosis—two ICD modalities. By releasing Fe^3+^ and tannic acid in melanoma cells, TPL@TFBF initiated Fenton reactions for lipid peroxidation (ferroptosis) while activating gasdermin D-mediated pyroptosis. The resultant DAMPs, including ATP and HMGB1, recruited DCs and enhanced CD8^+^ T cell priming. When combined with anti-PD-1 therapy, this strategy achieved complete tumor regression in 40% of treated cases, showcasing MNPs’ ability to convert immunologically “cold” tumors into “hot” lesions (112).

### 4.5 Exosome-based NPs

Nearly all cell types secrete exosomes, nanoscale vesicles measuring 40–160 nm in diameter. These vesicles play a pivotal role in intercellular communication by transporting bioactive molecules—including lipids, DNA, proteins, and various RNA species (such as microRNAs)—from donor to recipient cells (113). Ubiquitous in bodily fluids, exosomes are readily accessible through liquid biopsies, making them valuable sources of molecular information. Originating from late endosomes and multi-vesicular bodies (MVBs), exosomes are released into the extracellular space upon MVB fusion with the plasma membrane. Remarkably, they retain both the surface morphology and luminal content of their parent cells, including tumor cells (114). Upon reaching target cells, exosomes deliver their cargo either through membrane fusion or phagocytosis, thereby modulating recipient cell phenotypes.

#### 4.5.1 Therapeutic potential in oncology

While exosome-based cancer therapeutics require further clinical investigation, preclinical studies have demonstrated significant promise. Current immunotherapies, despite revolutionizing cancer treatment and improving survival rates in advanced cases, show limited efficacy against solid tumors compared to hematologic malignancies. This disparity stems from differential expression of immunoregulatory compounds that enhance immune cell interactions with hematological tumor cells (115).

The limited success of immunotherapy in solid tumors arises from several key challenges: The immunosuppressive and hypoxic TME impairs T cell proliferation, differentiation, and effector functions. Physical barriers (e.g., extracellular matrix, abnormal vasculature, cancer-associated fibroblasts) hinder immune cell infiltration and drug delivery while compromising T cell cytotoxicity. Immunosuppressive populations (MDSCs, Tregs, TAMs) within the TME actively suppress anti-tumor immunity (116).

For instance, while CTLA-4 inhibitors like ipilimumab enable 21% of patients to achieve 10-year survival and PD-1 inhibitors help 30% of advanced cancer patients survive beyond 5 years, these ICIs frequently cause immune-related adverse events (irAEs) (23). These limitations underscore the need for novel immunotherapeutic strategies with improved efficacy and safety profiles.

#### 4.5.2 Exosomes in tumor-immune crosstalk

As master regulators of intercellular signaling, exosomes mediate complex communication networks between tumor and immune cells. They facilitate immune cell activation through interimmune signaling while simultaneously presenting tumor-associated antigens to counteract immune evasion (82). Their unique ability to penetrate tumor ECM and resist TME-mediated suppression makes them particularly promising for overcoming cell therapy limitations (83).

#### 4.5.3 Advantages as drug delivery vehicles

The ideal drug delivery system must ensure safety, efficacy, and proper biodistribution. While various platforms (micelles, nanoparticles, liposomes) have been explored (117), exosomes offer distinct advantages: Natural biocompatibility: Low immunogenicity and high stability. Enhanced targeting: Bioengineered exosomes can deliver payloads (chemotherapeutics, RNAs) specifically to tumor cells (118). Biological barrier penetration: Innate ability to traverse biological obstacles while avoiding lysosomal degradation, improving delivery efficiency (92). Cargo protection: The vesicular structure shields contents from degradation, enhancing stability. This unique combination of properties positions exosomes as exceptionally promising vehicles for next-generation cancer therapeutics.

## 5 Mechanisms of action of nanodrug delivery systems in improving immunotherapeutic efficacy

### 5.1 Nanotechnology for adoptive cell therapy

Immunotherapy for melanoma encompasses a diverse array of approaches that fundamentally aim to harness and modulate the immune system’s capacity to recognize and eliminate tumor cells. These strategies—ranging from immune checkpoint inhibitors (ICIs) and tumor vaccines to nanomaterial-based delivery systems—are interconnected both mechanistically and clinically, as they often complement or enhance one another’s effects. For example, ICIs like anti-PD-1 and anti-CTLA-4 antibodies relieve inhibitory signals on T cells, thereby enabling a more robust immune response. Vaccine-based approaches aim to prime the immune system by presenting tumor-specific antigens, which can be further potentiated by nanocarriers that improve antigen delivery and immune activation. Nanoparticle platforms serve as versatile tools that can simultaneously deliver multiple immunomodulatory agents—such as cytokines, adjuvants, or genetic material—integrating the mechanisms of immune activation, suppression of immune evasion, and targeted delivery into a synergistic treatment paradigm. Clinically, combining these modalities can address the limitations of monotherapies, such as resistance or insufficient immune activation, providing a multifaceted attack against melanoma. Understanding how these strategies mechanistically interplay allows for the rational design of combination therapies that maximize therapeutic efficacy while minimizing adverse effects. ACT faces challenges in melanoma treatment due to immunosuppressive TMEs and limited T cell persistence. Nanotechnology is revolutionizing ACT by enhancing T cell expansion, reprogramming immune niches, and amplifying antitumor immunity through multifunctional platforms. Below we synthesize key advances in nanomaterial-engineered strategies to optimize ACT efficacy (**Table 5**).

**Table 5:** Nanotechnology for adoptive cell therapy.

Nanosystem	Approaches	Biological function	Reference
Dual-targeting M2-TAM-specific nanoparticles (M2NPs)	Conjugates α-M2pep peptides and SR-B1 ligands to deplete M2-TAMs, delivering siRNA	Reduces IL-10 and TGF-β secretion, enhances CD8+ T cell infiltration, induces tumor regression	(120)
Ginseng-derived extracellular vesicles (GDNPs)	Polarizes M2-TAMs to M1 phenotypes via TLR4/MyD88 signaling	Induces ROS-mediated apoptosis in melanoma cells, synergizes with ACT	(121)
Fraxinellone-loaded nanoemulsions (Frax NE)	Disrupts TAF activity and collagen deposition, reduces CXCL12/CXCR4 signaling	Enhances CD8+ T cell trafficking, synergizes with peptide vaccines to amplify tumor-specific immunity	(122)
Nanoscale artificial antigen-presenting cell (nano-aAPC) platform	Co-displays MHC class I/peptide complexes and anti-CD28 antibodies to stimulate T cell proliferation	Expands tumor-specific CD8+ T cells, generates high-affinity CTLs capable of lysing PD-L1+ melanoma cells	(123)
TME-responsive nanocarrier (MIT + CEL)	Co-delivers mitoxantrone (MIT) and celastrol (CEL) to induce dual ICD mechanisms	Induces DNA damage, calreticulin exposure, promotes DC activation and TIL recruitment, synergizes with ACT	(124)
Prussian blue nanoparticles (PBNPs)	Combined with near-infrared irradiation for PTT and ICD induction, paired with anti-CD137 antibodies	Upregulates co-stimulatory markers on DCs, amplifies CD8+ T cell functions, establishes immunological memory	(125)
Polymeric micelles loaded with sunitinib (SUNb-PM)	Blocks STAT3 phosphorylation in TAMs, delivers Trp2 peptide vaccine via LCP nanoparticles	Reverses VEGF-driven immunosuppression, increases CD8+ T cells, suppresses metastatic progression in melanoma	(126)

#### 5.1.1 Reprogramming immunosuppressive TMEs

##### 5.1.1.1 Targeting TAMs

Dual-targeting M2-TAM-specific NPs (M2NPs) were designed to silence immunosuppressive signals via siRNA delivery. By conjugating α-M2pep peptides and SR-B1 ligands, M2NPs selectively depleted M2-TAMs, reducing IL-10 and TGF-β secretion while enhancing CD8^+^ T cell infiltration. This remodeling of the TME led to 70% tumor regression in aggressive melanoma models (119, 120). Similarly, ginseng-derived extracellular vesicles (GDNPs) polarized M2-TAMs to pro-inflammatory M1 phenotypes via TLR4/MyD88 signaling, inducing ROS-mediated apoptosis in melanoma cells and synergizing with ACT (121).

##### 5.1.1.2 Combating stromal fibrosis:

Fraxinellone-loaded nanoemulsions (Frax NE) disrupted tumor-associated fibroblast (TAF) activity and collagen deposition. By reducing CXCL12/CXCR4 axis signaling, Frax NE enhanced intratumoral CD8^+^ T cell trafficking and synergized with peptide vaccines to amplify tumor-specific immunity (122).

#### 5.1.2 Enhancing T cell expansion and functionality

A nanoscale artificial antigen-presenting cell (nano-aAPC) platform was developed to rapidly expand tumor-specific CD8^+^ T cells. Co-displaying MHC class I/peptide complexes and anti-CD28 antibodies, nano-aAPCs stimulated robust T cell proliferation ex vivo, generating high-affinity CTLs) capable of lysing PD-L1+ melanoma cells. These nano-aAPC-primed T cells showed enhanced persistence and efficacy when combined with anti-PD-1 therapy (123).

#### 5.1.3 Inducing ICD for antigen priming

A TME-responsive nanocarrier co-delivering mitoxantrone (MIT) and celastrol (CEL) triggered dual ICD mechanisms: MIT induced DNA damage and calreticulin exposure, while CEL inhibited heat shock proteins to amplify DAMPs release. This strategy converted immunosuppressive TMEs into immunogenic niches, promoting DC activation and TIL recruitment, which synergized with ACT to induce tumor dormancy (124).

#### 5.1.4 Multimodal synergy with PTT

Prussian blue NPs (PBNPs) combined with near-infrared irradiation induced localized hyperthermia, eliciting ICD and upregulating co-stimulatory markers (CD80/86) on DCs. When paired with anti-CD137 antibodies, PBNP-PTT amplified CD8^+^ T cell effector functions and established immunological memory, achieving 90% suppression of melanoma recurrence (125).

#### 5.1.5 Combinatorial kinase inhibition and vaccine delivery

Polymeric micelles loaded with sunitinib (SUNb-PM) reversed VEGF-driven immunosuppression by blocking STAT3 phosphorylation in TAMs. Concurrent delivery of a Trp2 peptide vaccine via mannose-modified LCP NPs enhanced antigen cross-presentation. This dual approach increased tumor-infiltrating CD8^+^ T cells by 3-fold and suppressed metastatic progression in late-stage melanoma (126).

### 5.2 Nanotechnology for ICB therapy

ICB targeting PD-1/PD-L1 has revolutionized melanoma treatment, yet its efficacy remains limited by tumor immune evasion and immunosuppressive microenvironments. Nanotechnology offers innovative strategies to enhance ICB by improving drug delivery, amplifying ICD, and reprogramming the TME. Below we synthesize cutting-edge advances in nanoplatforms designed to synergize with ICB, addressing both primary and metastatic melanoma (**Table 6**).

**Table 6:** Nanotechnology for ICB therapy.

Nanosystem	Approaches	Biological function	Reference
Disulfide-crosslinked polyethyleneimine and trehalose sulfate nanoparticles (siPD-L1/pd)	Silencing of PD-L1 via RNA interference	Reverses T cell exhaustion, suppresses tumor growth, restores antitumor immunity in immunocompromised models	(115)
Tetrahedral framework nucleic acid immunoadjuvants (FNAIAs)	Co-loads CpG ODN and doxorubicin (DOX) for ICD induction	Activates DCs via TLR9 signaling, induces immunogenic cell death, triggers systemic antitumor immunity	(116)
Nano-hydroxyapatite (nHA)-incorporated hydrogel	Enhances PD-L1 expression and co-delivers PD-1 inhibitors	Promotes CD8+ T cell infiltration, polarization, blocks PD-1/PD-L1 interactions, inhibits tumor growth, enhances immune memory	(117)
Hydrazine/Cu/Fe/indocyanine green (TCFI) coordination nanocomplex	Induces ferroptosis via Fenton-like reactions, combined with anti-PD-1 therapy	Amplifies oxidative stress, increases CD8+ T cell infiltration, enhances IFN-γ secretion, suppresses tumor and metastases	(118)
Redox-responsive nanocarriers (MS-275 + V-9302)	Co-delivers histone deacetylase inhibitor and glutamine antagonist, exploits ROS for pyroptosis	Transforms “cold” tumors to “hot” tumors, enhances DC maturation and CTL infiltration, induces durable immune memory	(119)

#### 5.2.1 Targeted silencing of PD-L1 via RNA interference

Polymer-based NPs have been engineered to overcome PD-L1-mediated immune resistance. A study utilizing disulfide-crosslinked polyethyleneimine and trehalose sulfate NPs (siPD-L1/pd) demonstrated dual inhibition of PD-1/PD-L1 signaling and mTOR pathways. These NPs efficiently delivered PD-L1 siRNA to melanoma cells, silencing PD-L1 expression and reversing T cell exhaustion. In both immunocompetent and immunocompromised murine models, siPD-L1/pd NPs suppressed tumor growth and prolonged survival, highlighting their potential to restore antitumor immunity even in hosts with impaired immune systems (115).

#### 5.2.2 ICD induction through combinatorial nanotherapies

Tetrahedral framework nucleic acid immunoadjuvants (FNAIAs) represent a breakthrough in transdermal immunotherapy. Composed of CpG oligodeoxynucleotide (ODN)-loaded DNA nanostructures, FNAIAs penetrate tumor tissues to activate DCs via TLR9 signaling. When co-loaded with DOX, the resulting DFNAIA nanocomplex induces ICD, releasing tumor-associated antigens that synergize with CpG ODN to trigger systemic antitumor immunity. In melanoma models, DFNAIA combined with anti-PD-1 antibodies not only eradicated local tumors but also suppressed distant metastases, demonstrating its capacity to convert localized therapy into systemic immune activation (116).

#### 5.2.3 Remodeling the TME with PD-L1-enhancing NPs

Paradoxically, nanotechnology can also exploit upregulated PD-L1 for therapeutic benefit. nHA was shown to transcriptionally enhance PD-L1 expression in melanoma cells via calcium-sensing receptor signaling. Leveraging this phenomenon, researchers developed an injectable nHA-incorporated hydrogel that co-delivers PD-1 inhibitors. The hydrogel acts as a sustained-release depot, promoting CD8^+^ T cell infiltration and polarization while blocking PD-1/PD-L1 interactions. This strategy not only inhibited primary tumor growth but also established long-term immune memory, offering a dual-target approach to enhance ICB efficacy (117).

#### 5.2.4 Metal-based platforms for ferroptosis-ICB synergy

A hydrazine/Cu/Fe/indocyanine green (TCFI) coordination nanocomplex exemplifies nanotechnology’s capacity to integrate multiple therapeutic modalities. Under near-infrared irradiation, TCFI exhibits Fenton-like, peroxidase-, and glutathione oxidase-like activities, inducing lipid peroxidation and ferroptosis. Concurrently, it alleviates hypoxia and depletes glutathione to amplify oxidative stress. When combined with anti-PD-1 therapy, TCFI significantly increased tumor-infiltrating CD8^+^ T cells and IFN-γ secretion, achieving robust suppression of primary tumors, lung metastases, and recurrence. This work underscores the potential of metal-based nanoplatforms to synergize ferroptosis with ICB (118).

#### 5.2.5 Epigenetic-metabolic reprogramming for “cold” tumor conversion

In uveal melanoma (UVM), a redox-responsive nanocarrier co-delivering histone deacetylase inhibitor MS-275 and glutamine antagonist V-9302 was developed to overcome ICB resistance. The NPs exploit UVM’s high ROS levels to trigger ROS storms and pyroptosis—a proinflammatory cell death pathway. This approach transformed immunologically “cold” UVM into “hot” tumors by enhancing DC maturation and CTL infiltration. Combined with anti-PD-1 therapy, the system induced durable immune memory and suppressed metastatic progression, providing a blueprint for overcoming ICB resistance in refractory melanomas (119).

### 5.3 Nanotechnology for cancer vaccines

Nanotechnology is redefining melanoma vaccine design through innovative strategies that enhance antigen delivery, amplify immune activation, and subvert tumor immunosuppression. By leveraging biomimetic engineering, viral-inspired platforms, and combinatorial therapies, nanovaccines are overcoming the limitations of conventional immunotherapy to elicit robust and durable antitumor responses (**Table 7**).

**Table 7:** Nanotechnology for cancer vaccines.

Nanosystem	Approaches	Biological function	Reference
Erythrocyte membrane-camouflaged nanoparticles	PLGA core loaded with reduction-sensitive antigen peptides, cloaked in mannose-modified erythrocyte membranes	Enhances lymphatic targeting, DC uptake, antigen cross-presentation, and CTL activation, suppresses melanoma progression	(127)
Golgi-PD-L1-deficient exosome hybrid membrane-coated nanoparticles (GENPs)	Mimics natural exosome trafficking, delivers tumor antigens to lymph node DCs	Evades PD-L1 immune checkpoints, synergizes with anti-PD-L1 therapy, reduces tumor recurrence and metastasis	(128)
Cowpea mosaic virus (CPMV) nanoparticles	Selectively targets metastatic niches in the lung, recruits neutrophils and NK cells	Establishes premetastatic immunity, prevents metastatic growth, eradicates established lesions	(129)
Inactivated CPMV (inCPMV) platform	Engages TLR2/4 signaling, synergizes with OX40 agonistic antibodies	Reprograms immunosuppressive microenvironments, amplifies CD8+ T cell infiltration, induces systemic antitumor immunity	(130)
Co-assembled 2′-fluorinated CpG and Obsl1 neoantigen	Co-assembles CpG and melanoma neoantigen for precise antigen delivery	Achieves high antigen loading, targets DCs in lymph nodes, induces neoantigen-specific CTLs and long-term immunity	(131)
Poly(β-amino ester) (PBAE) nanovaccines	Co-encapsulates Trp-2 antigen and TLR4 agonist MPLA, combined with PD-L1 blockade antibodies	Primed DCs, counteracted T cell exhaustion, converted cold melanomas to hot lesions, achieved tumor regression	(132, 133)
Sunitinib-loaded nanoparticles (SUN-NPs)	Normalizes vasculature and depletes MDSCs, primes the TME for vaccination	Enhances CTL infiltration, synergizes with Trp-2/GalCer nanovaccines to overcome immunosuppressive barriers	(134)
Lipid nanoparticle vaccine (GalCer + Melan-A/gp100)	Co-delivers α-galactosylceramide (GalCer), melanoma antigens, and adjuvants (CpG/MPLA)	Activates iNKT cells, amplifies DC activation and antigen-specific CTL responses, reduces tumor volume and recurrence	(135)

#### 5.3.1 Biomimetic nanovaccines for targeted immune activation

Erythrocyte membrane-camouflaged NPs exemplify the power of bioinspired design in vaccine delivery. These platforms utilize PLGA cores loaded with reduction-sensitive antigen peptides, cloaked in mannose-modified erythrocyte membranes to enhance lymphatic targeting and DC uptake. Preclinical studies demonstrated superior antigen cross-presentation and CTL activation compared to non-coated systems, achieving 60% suppression of metastatic melanoma progression (127). Building on this concept, Golgi-PD-L1-deficient exosome hybrid membrane-coated NPs (GENPs) mimic natural exosome trafficking to deliver tumor antigens directly to lymph node DCs. By evading PD-L1-mediated immune checkpoints, GENPs synergized with anti-PD-L1 therapy to reduce tumor recurrence and distant metastasis, highlighting their dual role as antigen carriers and immune checkpoint modulators (128).

#### 5.3.2 Viral vector platforms harnessing innate immunity

The cowpea mosaic virus (CPMV) has emerged as a potent immunostimulant due to its intrinsic ability to activate pathogen recognition receptors. CPMV NPs selectively target S100A9-expressing metastatic niches in the lung, recruiting neutrophils and natural killer cells to establish premetastatic immunity. This approach not only prevented metastatic outgrowth but also eradicated established lesions, showcasing its potential as a prophylactic and therapeutic vaccine (129). A modified inactivated CPMV (inCPMV) platform further advanced safety without compromising efficacy. By engaging TLR2/4 signaling, inCPMV reprogrammed immunosuppressive microenvironments and synergized with OX40 agonistic antibodies to amplify CD8^+^ T cell infiltration, inducing systemic antitumor immunity even in non-treated tumors (130).

#### 5.3.3 Self-assembling systems for precision antigen delivery

A breakthrough in carrier-free vaccine design was achieved through the spontaneous co-assembly of 2′-fluorinated CpG (2′F-CpG) and the melanoma neoantigen Obsl1. This system achieved near-complete antigen loading efficiency and leveraged size-dependent lymphatic drainage to target DCs in lymph nodes. The resulting nanovaccine triggered a 10-fold increase in neoantigen-specific CTLs compared to soluble antigens, establishing long-term immune memory against tumor rechallenge. This platform underscores the potential of minimalist nanovaccines for personalized immunotherapy (131).

#### 5.3.4 Combinatorial strategies to reprogram the TME

Poly(β-amino ester) (PBAE) nanovaccines exemplify the synergy between antigen delivery and immune checkpoint modulation. By co-encapsulating Trp-2 antigen and TLR4 agonist MPLA, PBAE NPs primed DCs while PD-L1 blockade antibodies counteracted T cell exhaustion. This dual strategy converted immunologically “cold” melanomas into “hot” lesions, achieving complete regression in 40% of treated cases (132, 133). In fibrotic melanomas, sunitinib-loaded NPs (SUN-NPs) normalized dysregulated vasculature and depleted MDSCs, effectively “priming” the TME for subsequent vaccination. When combined with Trp-2/GalCer nanovaccines, SUN-NPs increased CTL infiltration 8-fold, demonstrating how nanotechnology can sequentially dismantle immunosuppressive barriers (134).

#### 5.3.5 Lipid-based systems engaging innate and adaptive immunity

A lipid NP vaccine co-delivering α-galactosylceramide (GalCer) with melanoma antigens (Melan-A/gp100) and adjuvants (CpG/MPLA) leveraged the unique biology of invariant natural killer T (iNKT) cells. The lipid bilayer stabilized GalCer presentation to iNKT cells, which in turn activated DCs and amplified antigen-specific CTL responses. This approach reduced tumor volume by 90% and established protective immunity against recurrence, outperforming GalCer-free formulations (135).

### 5.4 Nanotechnology for cytokine therapy

Cytokine therapy, particularly involving interleukin-12 (IL-12), has long been explored for its potential to activate antitumor immunity in melanoma. However, clinical translation has been hampered by systemic toxicity, short half-life, and immunosuppressive TMEs. The development of the CaCO3-polydopamine-polyethyleneimine (CPP) NP platform exemplifies how nanotechnology can overcome these limitations by enabling targeted delivery, spatiotemporal control, and multimodal synergy (136).

## 6 Clinical trials

Nanotechnology has rapidly advanced over the past few decades, leading to the development of numerous NP-based cancer therapies, including immunotherapies for melanoma. To date, there are over 80 novel cancer nanomedicines being evaluated in more than 200 clinical trials globally, with several FDA-approved nanomedicines, such as liposomal formulations of chemotherapeutics like Doxil^®^ (NCT01846611, Phase 3) and Marqibo^®^ (FDA approved), successfully navigating clinical development (137–140). However, nano-immunotherapy for melanoma is still in the early stages of exploration. Current clinical trials focus on utilizing nanotechnology to enhance ICB, stimulate immune responses, and improve drug delivery in melanoma treatment.

For example, nanobodies have emerged as promising candidates for antigen-targeting domains in chimeric antigen receptor (CAR)-T cells. Clinical trials have shown that nanobody-based CAR-T therapies exhibit comparable efficacy to traditional single-chain variable fragment (scFv)-based CAR-T cells in treating hematologic malignancies (141, 142), and similar approaches are now being adapted for melanoma treatment (143, 144). Other promising clinical trials involve the use of NPs for targeted delivery of immunotherapeutic agents, such as ICIs and cancer vaccines, in combination with other therapies like cyclophosphamide and pembrolizumab (145–148). Additionally, ongoing trials are exploring the use of NP-based vaccines, such as Maveropepimut-S, and lipid NPs encapsulating mRNA to further augment immune responses against melanoma (149). As nano-immunotherapies continue to progress in clinical trials, they hold the potential to revolutionize melanoma treatment by improving specificity, enhancing immune activation, and overcoming challenges within the TME. While nanotechnology holds significant promise for enhancing melanoma immunotherapy, translating these advances into clinical practice is impeded by manufacturing challenges, safety concerns, tumor heterogeneity, and regulatory hurdles. Addressing these barriers requires interdisciplinary efforts to optimize nanoparticle design, establish standardized protocols, ensure safety and efficacy through comprehensive preclinical and clinical studies, and develop cost-effective manufacturing solutions. The clinical translation of nanomedicines for melanoma faces multiple challenges, including scalability and manufacturing complexities (e.g., difficulties in large-scale production of biomimetic nanoparticles under GMP standards), safety and immunogenicity concerns (such as unintended immune responses and limited long-term toxicity data), and biological barriers like tumor heterogeneity and the immunosuppressive TME. Additional hurdles include maintaining *in vivo* stability and targeting specificity, navigating regulatory and clinical trial obstacles (due to a lack of standardized biomarkers and complex combination therapies), and high production costs that limit accessibility. Addressing these challenges is critical for advancing effective and widely applicable nanomedicine-based treatments.

## 7 Discussion and prospects

The integration of nanotechnology into melanoma immunotherapy has significantly advanced the field, enabling more precise treatments and overcoming traditional barriers. Key developments in lipid-based, protein-based, and metallic nanoparticles (MNPs), as well as nanovaccines and cytokine delivery systems, have shown great promise in reprogramming the immunosuppressive TME, boosting antigen-specific immunity, and enhancing the efficacy of conventional therapies. LNPs have evolved into multifunctional platforms, capable of silencing immunosuppressive signals while delivering therapeutic agents. For example, LNPs incorporating immune checkpoint modulators or STING agonists can both inhibit TGF-β and deliver chemotherapeutic agents, allowing for real-time monitoring and effective treatment. Protein-based NPs (PNPs) utilize biological specificity to neutralize tumor-derived immunosuppressive ligands, promoting immune cell infiltration and stimulating long-lasting immune responses against both localized and metastatic melanoma. MNPs further enhance therapeutic potential by combining CDT with photothermal ablation, inducing ICD and activating systemic immunity through pathways like cGAS-STING. Additionally, advancements in ACT and nanovaccines, such as artificial nano-aAPCs and neoantigen vaccines, offer highly targeted and efficient strategies for tumor-specific T cell expansion and improved vaccine delivery. These innovations signal a shift towards personalized, multimodal immunotherapies tailored to the unique characteristics of each patient’s tumor.

Despite these breakthroughs, critical challenges impede clinical translation. Scalable manufacturing remains a bottleneck, particularly for biomimetic systems requiring complex synthesis protocols, such as erythrocyte membrane-coated NPs or viral-inspired platforms. Immunogenicity and off-target effects also pose risks, as seen with protein-based NPs that may inadvertently trigger anti-carrier immune responses. Furthermore, the heterogeneity of melanoma subtypes demands adaptable platforms capable of addressing diverse mutational landscapes and TME variations. Current strategies often lack the dynamic responsiveness needed to evolve alongside tumor adaptation, limiting their long-term efficacy. Looking ahead, the field must prioritize intelligent nanoplatforms that marry precision delivery with real-time adaptability. Stimuli-responsive designs—such as pH- or enzyme-triggered release systems—could enhance spatiotemporal control over therapeutic payloads, minimizing systemic toxicity. Integrating diagnostic agents like MRI-active iron oxides or NIR dyes into NPs would advance theranostic applications, enabling clinicians to monitor treatment responses and adjust regimens dynamically. Emerging technologies such as computational protein design and AI-driven antigen prediction could further accelerate the development of personalized nanovaccines, tailoring therapies to individual neoantigen profiles. Additionally, interdisciplinary collaboration will be essential to standardize manufacturing processes and validate long-term safety in immunocompetent models, ensuring compliance with Good Manufacturing Practice (GMP) standards. Limitations includes:

1. Scalability and Manufacturing Challenges: Difficulties in producing complex nanomaterials consistently and cost-effectively at a clinical scale, especially for biomimetic and multifunctional platforms.
2. Safety and Toxicity Concerns: Potential immunogenicity, off-target effects, accumulation in non-target tissues, and long-term toxicity remain significant hurdles that require thorough evaluation in preclinical and clinical models.
3. Heterogeneity of Melanoma and TME: Variability among patients’ tumors and TMEs can limit the broad applicability of certain nanoplatforms, necessitating personalized or adaptable approaches.
4. Delivery Barriers: Biological barriers such as the mononuclear phagocyte system (MPS), rapid clearance, and the TME’s distinct features can impair nanoparticle accumulation and penetration.
5. Regulatory and Translational Barriers: Lack of standardized protocols, limited clinical translation, and the need for extensive safety data pose hurdles for regulatory approval and widespread clinical adoption.

The future of melanoma immunotherapy lies in embracing nanotechnology not as a standalone solution but as a synergistic enhancer of existing modalities. Combining nanoplatforms with ICIs, epigenetic modulators, or targeted therapies could create layered treatment ecosystems capable of outmaneuvering tumor evolution. For example, NPs delivering CRISPR-Cas9 systems to edit PD-1 in T cells *in vivo* might overcome resistance to anti-PD-1 antibodies, while MOFs loaded with ferroptosis inducers could amplify the effects of radiotherapy. Clinically, biomarker-driven trials using liquid biopsies or exosome profiling could identify patient subgroups most likely to benefit from specific nanotherapies, optimizing therapeutic outcomes.

## 8 Conclusion

Advantages and disadvantages for each type of nanoparticle (LNPs, polymeric NPs, protein-based NPs and MNPs) in terms of biocompatibility, biodegradability, stability and immune modulationLipid Nanoparticles (LNPs) are concluded as follow:

### 8.1 Lipid nanoparticles

Advantages: Biocompatibility: High, as lipid compositions often mimic biological membranes, resulting in minimal toxicity. Biodegradability: Good, since lipids can be naturally metabolized. Stability: Generally stable during storage, especially when formulated with protective lipids; capable of encapsulating hydrophilic and hydrophobic agents. Immune Modulation: Can incorporate adjuvants or targeting moieties to modulate immune responses effectively.

Disadvantages: Biocompatibility: Potential for activation of complement pathways, leading to infusion-related reactions if not properly optimized. Biodegradability: Lipid oxidation or hydrolysis can compromise stability over time. Stability: Prone to leakage of encapsulated agents and physical instability under certain conditions. Immune Modulation: May induce non-specific immune activation, leading to systemic side effects.

### 8.2 Polymeric nanoparticles

Advantages: Biocompatibility: Many polymers (e.g., PLGA, PCL) are biocompatible and approved for clinical use. Biodegradability: Designed to degrade into non-toxic monomers within biological environments. Stability: Excellent mechanical and chemical stability, allowing for controlled release profiles. Immune Modulation: Can be functionalized to target specific immune cells or deliver immunostimulatory agents selectively.

Disadvantages: Biocompatibility: Potential for residual monomers or degradation products to cause toxicity if not thoroughly processed. Biodegradability: Degradation rate varies with polymer composition and environment, which may impact timing of drug release. Stability: Susceptible to hydrolysis and premature degradation if not properly formulated. Immune Modulation: Risk of unintended immune activation or clearance by the mononuclear phagocyte system, reducing efficacy.

### 8.3 Protein-based nanoparticles

Advantages: Biocompatibility: High, as they are composed of naturally occurring proteins. Biodegradability: Readily degraded by proteases *in vivo*, minimizing long-term accumulation. Stability: Can be stabilized through cross-linking or surface modifications, though generally sensitive to environmental conditions. Immune Modulation: Intrinsic ability to engage immune receptors; can be designed as vaccines or immune stimulants.

Disadvantages: Biocompatibility: Potential for immunogenicity and unintended immune responses against the protein carrier. Biodegradability: Rapid enzymatic degradation may limit circulation time unless stabilized. Stability: Prone to denaturation or aggregation under certain storage or physiological conditions. Immune Modulation: May induce unwanted immune responses, including antibody formation against the carrier proteins.

### 8.4 Metallic nanoparticles

Advantages: Biocompatibility: Varies; gold nanoparticles are often well tolerated, but others (e.g., silver, copper) may pose toxicity concerns. Biodegradability: Generally inert and non-degradable; long-term accumulation may occur. Stability: Highly stable and resistant to chemical degradation, suitable for imaging and therapeutic functions. Immune Modulation: Can act as immune adjuvants or facilitate photothermal and radiotherapy synergistically.

Disadvantages: Biocompatibility: Potential toxicity if particles are not properly coated or purified, leading to oxidative stress or tissue accumulation. Biodegradability: Limited; long-term retention can cause adverse effects. Stability: Susceptible to aggregation and oxidation, which can affect safety and efficacy. Immune Modulation: May induce unintended immune responses or inflammation, especially if surface properties are not optimized.

Nanotechnology is redefining the rules of engagement in melanoma treatment, transforming once-insurmountable challenges into opportunities for innovation. By bridging gaps between targeted delivery, immune activation, and diagnostic precision, nanoplatforms are poised to shift the therapeutic paradigm from reactive tumor suppression to proactive immune control. Realizing this vision will require relentless focus on translational science—scaling production, validating safety, and fostering partnerships across academia, industry, and regulatory bodies. As these efforts converge, the day when melanoma becomes a manageable chronic condition draws nearer, marking a triumph of interdisciplinary science over one of oncology’s most formidable adversaries.

## Glossary

TME: tumor microenvironment
ICIs: Immune checkpoint inhibitors
UV: ultraviolet
EPR: enhanced permeability and retention
DC: dendritic cell
Tregs: regulatory T cells
MDSCs: myeloid-derived suppressor cells
PFS: progression-free survival
OS: overall survival
RFS: relapse-free survival
CAFs: cancer-associated fibroblasts
MES: mesenchymal
CPPs: cell-penetrating peptides
LNPs: Lipid-based nanoparticles
LCP: lipid-calcium-phosphate
l LPH: ipid-polyarginine-hyaluronic
PEG-EV-DOX: PEGylated extracellular vesicles
IL-13-LCL-SIM: IL-13-functionalized long-circulating liposomes
APCs: antigen-presenting cells
artificial antigen-presenting cells: nano-aAPCs, ICD, immunogenic cell death
PNPs: Polymeric nanoparticles
PAMP: pathogen-associated molecular pattern
TMV: tobacco mosaic virus
nHA: nano-hydroxyapatite
PNT: peptide nanotube
PDT: photodynamic therapy
FCS: fluorocarbon-modified chitosan
MNPs: Metallic-based nanoparticles
FMT: ferumoxytol
ZMS: Manganese-zinc sulfide
CDT: chemodynamic therapy
PTT: photothermal therapy
ROS: reactive oxygen species
DAMPs: damage-associated molecular patterns
rMMNs: Redox-responsive magnetic nanocarriers
ICB: Immune checkpoint blockade
FNAIAs: framework nucleic acid immunoadjuvants
ODN: oligodeoxynucleotide
DOX: doxorubicin
UVM: uveal melanoma
ACT: Adoptive cell therapy
M2NPs: M2-TAM-specific nanoparticles
Frax NE: Fraxinellone-loaded nanoemulsions
nano-aAPC: nanoscale artificial antigen-presenting cell
MIT: mitoxantrone
CEL: celastrol
TIL: tumor-infiltrating lymphocyte
PBNPs: Prussian blue nanoparticles
SUNb-PM: Polymeric micelles loaded with sunitinib
CTL: cytotoxic T lymphocyte
GENPs: Golgi-PD-L1-deficient exosome hybrid membrane-coated nanoparticles
CPMV: cowpea mosaic virus
inCPMV: inactivated
2′F-CpG: 2′-fluorinated CpG
SUN-NPs: sunitinib-loaded nanoparticles
GalCer: galactosylceramide
iNKT: invariant natural killer T
IL-12: interleukin-12
CPP: CaCO3-polydopamine-polyethyleneimine
NP: nanoparticle
CAR: chimeric antigen receptor
scFv: single-chain variable fragment
MNPs: Metallic nanoparticles
GMP: Good Manufacturing Practice
MOFs: metal-organic frameworks
